# P02-026 - Model-based characterization of the PKPD relationship for canakinumab in CAPS: a step towards personalized

**DOI:** 10.1186/1546-0096-11-S1-A133

**Published:** 2013-11-08

**Authors:** A Gautier, P Lowe, A Skerjanec, P McKernan, O Luttringer, M Fink

**Affiliations:** 1 Novartis Pharma AG, Basel, Switzerland

## Introduction

Canakinumab is a high-affinity fully human monoclonal antibody of the IgG1/k isotype, designed to bind and functionally neutralize the bioactivity of IL-1β, which is recognized as one of the principal pro-inflammatory cytokines in cryopyrin associated periodic syndromes (CAPS).

## Objectives

The objectives of the study were to describe the kinetics of canakinumab and dynamics of binding IL-1β in CAPS patients; to determine if these are different in 2- and 3-year-old children versus older children and adults; and to explore the impact of CAPS phenotype (Muckle-Wells Syndrome [MWS], Familial Cold Autoinflammatory Syndrome [FCAS], Neonatal-Onset Multisystem Inflammatory Disease [NOMID]) on the kinetics of canakinumab and dynamics of binding to IL-1β.

## Methods

A pharmacokinetics (PK)-binding model was used to describe the kinetic and binding parameters of canakinumab and IL-1β in CAPS patients, and in other populations relative to CAPS. The subgroup of 7 CAPS patients who were 2 and 3 years of age at baseline was also compared to the overall CAPS population.

## Results

The 7 CAPS patients did not show any difference in terms of PK. However, they showed a higher IL-1β turnover including IL-1β clearance and production. IL-1β levels were linked with the severity of the CAPS phenotype. In the pediatric population, MWS and especially NOMID patients had higher concentrations of the inert canakinumab/IL-1β complexes after administration of canakinumab, indicating more cytokine in the body to be captured.

## Conclusion

Correlation with clinical responses suggested that these increased levels of IL-1β may explain why younger and NOMID phenotype patients require higher doses or escalation to higher doses.

## Disclosure of interest

A. Gautier Shareholder of: Novatis Pharma AG, Employee of: Novatis Pharma AG, P. Lowe Shareholder of: Novartis Pharma AG, Employee of: Novartis Pharma AG, A. Skerjanec Employee of: Novartis Pharma AG, P. McKernan Shareholder of: Novartis Pharma AG, Employee of: Novartis Pharma AG, O. Luttringer Shareholder of: Novartis Pharma AG, Employee of: Novartis Pharma AG, M. Fink: None Declared.

